# *Sambucus ebulus* (Elderberry) Fruits Modulate Inflammation and Complement System Activity in Humans

**DOI:** 10.3390/ijms24108714

**Published:** 2023-05-13

**Authors:** Yoana Kiselova-Kaneva, Milka Nashar, Bogdan Roussev, Ayshe Salim, Minka Hristova, Pawel Olczyk, Katarzyna Komosinska-Vassev, Ivayla Dincheva, Ilian Badjakov, Bistra Galunska, Diana Ivanova

**Affiliations:** 1 Department of Biochemistry, Molecular Medicine and Nutrigenomics, Faculty of Pharmacy, Medical University “Prof. Dr. Paraskev Stoyanov”, 9002 Varna, Bulgaria; milka.nashar@mu-varna.bg (M.N.); bogdanroussev@gmail.com (B.R.); ayshe.salim@mu-varna.bg (A.S.); galunska@mu-varna.bg (B.G.); divanova@mu-varna.bg (D.I.); 2 Department of Physiology and Pathophysiology, Faculty of Medicine, Medical University “Prof. Dr. Paraskev Stoyanov”, 9002 Varna, Bulgaria; hristova_minka@mu-varna.bg; 3 Department of Community Pharmacy, Faculty of Pharmaceutical Sciences, Medical University of Silesia in Katowice, 40-055 Katowice, Poland; polczyk@sum.edu.pl; 4 Department of Clinical Chemistry and Laboratory Diagnostics, Faculty of Pharmaceutical Sciences in Sosnowiec, Medical University of Silesia in Katowice, 40-055 Katowice, Poland; kvassev@sum.edu.pl; 5 AgroBioInstitute, Agricultural Academy, 8 Dragan Tsankov Blvd., 1164 Sofia, Bulgaria; ivadincheva@yahoo.com (I.D.); ibadjakov@gmail.com (I.B.)

**Keywords:** herbs, Sambucus, anthocyanins, immunomodulatory, inflammation, complement, intervention

## Abstract

*Sambucus ebulus* (SE) fruits are used for immune stimulation and amelioration of gastrointestinal inflammatory conditions. Currently, there is no scientific evidence of their effects on various aspects of the immune response mechanisms in humans. The purpose of this study was to evaluate the immunomodulatory potential of SE fruit infusion intake in healthy humans. Anthocyanin content was determined with UPLC-ESI-MS/MS. Fifty-three volunteers enrolled in a 4-week SE infusion intake intervention. Blood count, serum total protein, Interleukin 1 beta (IL-1β), Interleukin 6 (IL-6), Tumor Necrosis Factor Alpha (TNFα), High-sensitivity C-reactive protein (hs-CRP), C3, and C4 levels were measured on automatic analyzers, and Interleukin 8 (IL-8) was measured manually with an ELISA kit. Cyanidin-3-O-galactoside (48.15 mg/g DW), followed by cyaniding-3-sambubioside (43.41 ± 1.07 mg/g DW), were the most abundant anthocyanins in SE samples. A significant decrease in total protein (2.82%), IL-6 (20.15%), TNFα (5.38%), IL-8 (5.50%), C3 (4.16%), and C4 (14.29%) was established in the whole group. Total protein, IL-8, TNFα, and C4 decreased in women (3.11%, 4.76%, 5.09%, and 11.11%), and IL-6 decreased (40.61%) in men. Hb (1.20%) and hematocrit (1.55%) levels decreased in the whole group and in the women group (1.61% and 2.20%). SE fruits exert immune-modulatory activity as revealed by decreased pro-inflammatory status and complement activity markers in healthy volunteers after a 4-week intervention.

## 1. Introduction

Nowadays, there is a growing interest in the usage of medicinal plants in the prevention and treatment of various disorders, alone or as complementary remedies. Millennial knowledge about the application of herbs, aromatic spices, and medicinal plants has been preserved, and the fast development of biomedical sciences provided opportunities for elucidation of the mechanisms behind their healing properties.

Based on empirical data from traditional medicine, many investigations revealed the potential of medicinal plants and their active compounds to inhibit inflammation and modulate the immune response through multiple mechanisms [1,2,3,4,5,6]. In light of the current challenges, e.g., those raised by the COVID-19 pandemic, there is a growing interest in a treatment with the potency to ameliorate the host’s defense since disease severity very often is not only a result of direct viral cytotoxicity but rather of an extremely powerful host reaction, including cytokine storm, causing magnified viral invasion and resulting in a destructive inflammatory response [7].

Elderberries (*Sambucus* sp.) are some of the most commonly used medicinal plants. Traditionally, the most popular is the usage of their well-ripened fruits, and less frequently, their leaves, flowers, and roots are used [8,9]. A growing research interest in the antiviral potential of *S. ebulus* and *S. nigra* fruits has been observed. A study reported the inhibitory capacity of wild elderberry fruit extracts on the receptor binding domain in the SARS-CoV-2 spike protein [10]. Elderberry supplementation, particularly *S. nigra* preparations, has already been recommended for the early course of COVID-19 disease treatment [11].

A significant amount of scientific data in recent decades reveals the role of phytochemical composition in the biological effects of medicinal plants. Fruits of *S. ebulus* (SE) are rich in polyphenols, anthocyanins, phytosterols, triterpenes, tannins, iridoid glycosides, cardiac glycosides, derivatives of caffeic acid (such as volatile substances), chlorogenic acid, ursolic acid, and lectins [12,13,14,15,16,17,18]. Elderberry extracts exert antioxidant activity in vitro [19,20,21]. Our previous studies on cell culture models have revealed anti-inflammatory [22,23,24] and antioxidant defense stimulating capacities [20] and cytoprotective [24] properties of SE fruit extracts in oxidative and inflammatory stimulated preadipocytes and macrophages. In addition, many other biological effects of *S. ebulus* fruits on cell cultures and experimental animals were reported related to antimicrobial [25,26], anticancer [27], anti-inflammatory [28], antidepressant [29], antigiardial [30], scolicidal [31], and neuroprotective [32] properties of the plant. A remarkable number of studies revealed the antiviral activity of elderberries. Zahmanov et al. [33] established the anti-herpes simplex activity of *S. ebulus* flavonoid glycosides. The anti-influenza virus activity of the elderberry fruit preparations has been demonstrated in cell culture studies as well as in in vitro studies. *Sambucus nigra* fruit juice and its primary anthocyanin cyanidin 3-glucoside, also found in *S. ebulus* [34], inhibited Human Influenza A (H1N1) infection [35,36]. In addition, studies revealed the activity of *S. nigra* against feline immunodeficiency virus [37] and against human respiratory viruses [38].

Evidence about the beneficial effects of Sambucus sp. plants is obtained from human interventional studies. In our previous interventional study, the *S. ebulus* fruits were found to improve lipid profile in healthy volunteers [39]. Some studies show that supplementation with *Sambucus nigra* was found to substantially reduce upper respiratory symptoms in case of viral infections, such as the common cold and influenza [40,41,42]. *S. ebulus* fruit extract has been found to be effective in treating paederus dermatitis in humans, exerting anti-inflammatory, wound healing, and analgesic effects [43].

Here, we present a human interventional study with fruit infusion from *Sambucus ebulus* L., which is a plant with a long history of use in the traditional medicine of the Balkans [8]. The most popular usage of *S. ebulus* fruits in Bulgarian folk medicine is for immune stimulation in the autumn/winter period and for the amelioration of gastrointestinal inflammatory conditions [20]. There are also indications for its use in the case of rheumatoid arthritis and for hematopoiesis stimulation [20]. Many studies are focused on the anti-inflammatory properties of *S. ebulus* leaves and flowers [44], and the fruits still remain incompletely studied, both in regard to their phytochemical composition and biological activity. Another research group in Bulgaria has obtained data about some SE metabolites (amino acids, organic acids, and secondary metabolites) and anti-herpes simplex activity of SE flavonoid glycosides [33]. Currently, there is no scientific evidence on the effect of the fruit infusion from *S. ebulus* on various aspects of the immune response and the mechanisms of this effect in humans. For this reason, the purpose of this study was to evaluate the effect of *S. ebulus* fruit infusion on pro-inflammatory cytokine levels and on two components of the complement cascade in relationship with the peripheral blood immune cells profile. As anthocyanins are considered to be among the most abundant phytochemicals in elderberry fruits, an analysis of anthocyanin content was performed to support observed biological activities.

## 2. Results

### 2.1. Phytochemical Analysis of S. ebulus Fruit Infusion

Six different cyanidin-3-O-glycosides were quantitatively analyzed in SE water infusion (Table 1). The most abundant among analyzed compounds was cyanidin-3-O-galactoside, present in a concentration of 48.15 mg/g DW, followed by cyaniding-3-sambubioside, found to be in a concentration of 43.41 ± 1.07 mg/g DW.

### 2.2. Biochemical and Hematological Analysis

**Figure 1:**
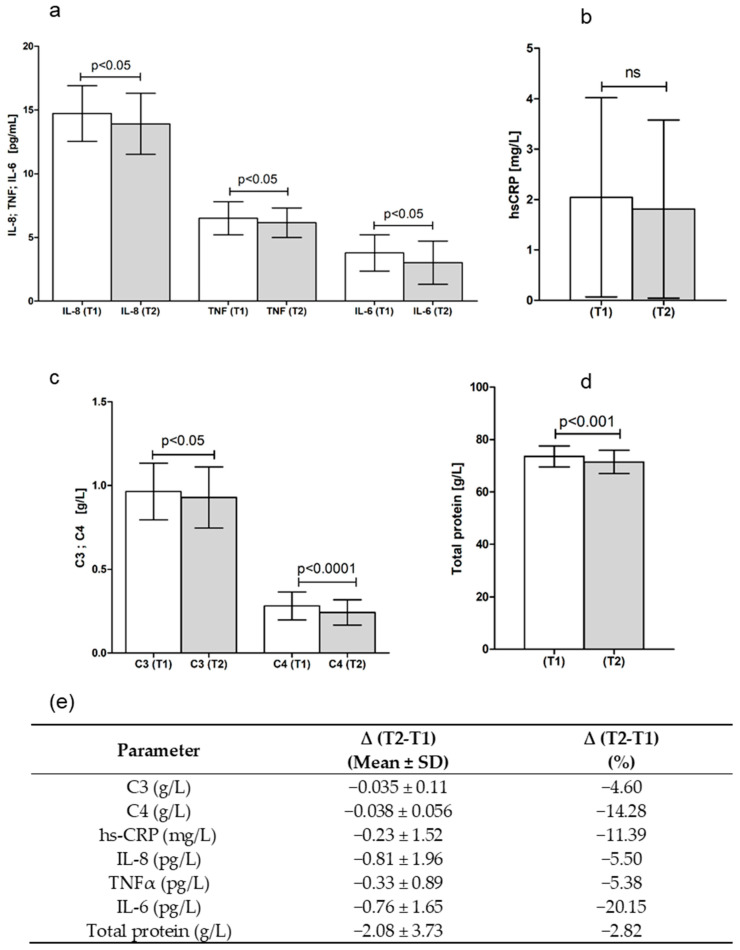
Biochemical parameters before and after intervention with *S. ebulus* fruit tea: (**a**) IL-6, IL-8, and TNFα before and after intervention; (**b**) hs-CRP before and after intervention; (**c**) C3 and C4 before and after intervention; (**d**) total protein before and after intervention; and (**e**) numerical change in studied parameters.

In order to study the possible anti-inflammatory potential of *S. ebulus* fruits, serum levels of inflammatory markers High-sensitive C-reactive protein (hs-CRP), Interleukin 1 beta (IL-1β), Interleukin 6 (IL-6), Tumor necrosis factor alpha (TNFα), and Interleukin 8 (IL-8) were measured before and after the intervention period. All of these parameters were decreased at the end of the intervention (Figure 1), representing an improved inflammatory status as a result of *S. ebulus* tea intake. The change was significant for IL-6 (20.15%, *p* < 0.05), TNFα (5.38%, *p* < 0.01), and IL-8 (5.50%, *p* < 0.01). In addition, total protein levels also decreased significantly by 2.82% (*p* < 0.001) in the whole group after the intervention. The analysis of the results, according to the gender of the participants, revealed a significant decrease in total protein, IL-8, and TNFα in women (3.11%, *p* < 0.001, 4.76%, *p* < 0.05, and 5.09%, *p* < 0.05, respectively) and of IL-6 (40.61%, *p* < 0.05) in men (Table 2). IL-1β was under the detection limit in most of the samples.

C3 and C4 levels in serum were measured in order to determine the possible effect of SE fruit tea intake on complement system activity (Figure 1). A significant decrease both in C3 and C4 was detected (4.16%, *p* < 0.05 and 14.29%, *p* < 0.001, respectively) in the whole group. Gender stratification showed a significant C4 decrease in women only (11.11%, *p* < 0.001) (Table 2), which was probably due to the fact that this group had more participants compared with the male group, and interindividual variations exceeded the difference before and after the tea intake.

The possible effects of *S. ebulus* fruit tea intake on host defense were studied by undertaking peripheral blood cell counts. With a borderline significance, the percentage of lymphocytes was increased in the whole group by 1.91% and in the women group by 5.42% (Table 2 and Table 3).

Measurement of hematological parameters showed a slight but significant decrease for Hb (1.20%, *p* < 0.05) and hematocrit (1.55%, *p* < 0.01) in the whole group after the intervention (Table 3). Gender stratification showed this change is sex-dependent, as these parameters were decreased in the women group only (1.61%, *p* < 0.01 and 2.20%, *p* < 0.001, respectively), where a slight, nonsignificant decrease in RBC was also detected. Bearing in mind the variations in hematological parameters depending on the menstrual cycle phases [45,46], we analyzed and compared levels of RBC, Hb, and hematocrit before and after *S. ebulus* intervention between two subgroups—one included men and women in menopause and the other with females in fertile age (Table 4).

In order to check how changes in inflammatory cytokines and C3 and C4 (Δ (T2-T1) depend on each other and hematologic counts, we performed a correlation analysis. Table 5 contains statistically significant values only.

## 3. Discussion

In recent decades, there has been a growing scientific interest in the immunomodulatory properties of medicinal plants. Many research reports have revealed the key role of phytochemicals such as flavonoids, polysaccharides, lactones, alkaloids, etc., for the effects of plant extracts on the immune system in the prevention and supplementary treatment of infections and inflammation-related conditions [47,48].

In light of the current challenges raised by the COVID-19 pandemic, there is a growing interest in a treatment with the potency to ameliorate the host’s defense. Very often, disease severity is not only a result of direct viral cytotoxicity but rather of an extremely powerful host reaction, including cytokine storm, causing magnified viral invasion and resulting in a destructive inflammatory response [7].

*Sambucus ebulus* is used in folk medicine for immune stimulation in the autumn/winter period and for the amelioration of gastrointestinal inflammatory conditions [20]. There are also indications for its use in the case of rheumatoid arthritis and for hematopoiesis stimulation [20]. This study was undertaken in order to obtain scientific data about the immune-modulation properties of *S. ebulus* fruits that can support their usage in traditional medicine and for functional food development. We studied immune modulatory activity on the level of inflammatory cytokine production, complement system activity, and blood cell numbers. In addition, UPLC-ESI-MS/MS analysis revealed the most abundant anthocyanins in the sample.

Anthocyanins are water-soluble plant pigments providing the red, purple, blue, or black color of respective organs. In this study, six different cyanidin-3-O-glycosides were quantitatively analyzed in *S. ebulus* fruit infusion. The most abundant anthocyanin among analyzed compounds was cyanidin-3-O-galactoside, present in a concentration of 48.15 mg/g DW, followed by cyanidin 3-sambubioside, found to be in a concentration of 43.41 ± 1.07 mg/g DW. Cyanidin-3-O-galactoside has been reported to be present in other purple-colored berries, such as Vaccinium species [49] and *Aronia melanocarpa* fruits [50], but not in elderberries. Instead, cyanidin-3-O-glucoside, which was established in considerably lower amounts here (32.62 ± 1.22 mg/g DW), has been most often reported for *S. nigra* and other Caprifoliaceae berries [51,52,53]. Cyanidin 3-sambubioside, which was also established to be most abundant among measured anthocyanins, has been reported in other Sambucus species, including *S. ebulus* [52,53]. Infusion preparation also led to the extraction of 10.82 mg/g DW of cyanidin-3-O-arabinoside and 1.81 mg/g DW of cyanidin-3-O-xyloside. These two anthocyanins are also contained in blueberries [49,54], chokeberries (Aronia) [55], and black and other berries [56]. Our recent study on SE fruit tea presents detailed data about its phytochemical composition in regard to 33 polyphenolic compounds (hydroxycinnamic acids, flavonol glucosides, stilbenes, proanthocyanidin mono-, and di and trimers). 5-O-caffeoylquinic acid, 3-*p*-cumaroylquinic acid, quercetin-3-O-galactosyde, *trans*-resveratrol-3-O-glucoside, epicatechin, and B-type procyanidin trimers were the most abundant compounds. Furthermore, in vitro antioxidant activity of the SE tea was established [18]. The established significant decreases in studied cytokines (IL-1β, IL-6, IL-8, and TNFα), the acute phase inflammatory protein hs-CRP, and total protein (Figure 1) are in support of the data of folk medicine application of *S. ebulus* fruits in case of inflammation-related disorders. Although IL-1β levels were under the detection limit in 46 out of 51 individuals, it was not significantly increased in the remaining 5 individuals. A significant decrease in total protein, IL-8, and TNFα was established in women and in IL-6 in men. The immune system is a complex network of molecules, cells, and tissues working in a coordinated manner in order to recognize and eliminate infectious agents, malignant and transformed cells, and other foreign and harmful antigens. Various exogenous factors can modulate the function of the immune system by inhibiting or stimulating components of innate or adaptive immune response. As part of the immune response, inflammation is an important protective response against pathogens, injury, or damaged cells. It is controlled by various biologically active molecules, including cytokines, chemokines, complement proteins, growth factors, eicosanoids, and peptides [57]. Cytokines play a key role in signal transduction pathways related to the regulation and modulation of inflammation and immune response. They work in a complex signal network, and their functions very often overlap. Cytokines have diverse biological effects on different types of cells, such as differentiation, proliferation, and production of other cytokines [58,59]. In this regard, viral infections, for example, are well known to be associated with increased cytokine production. The coronavirus infections that have recently gained importance are also described to be accompanied by enhanced secretion of cytokines, such as IL-1β, IL-6, and IL-8 [60].

Data from our previous studies revealed that *S. ebulus* fruit extracts and fractions have the potential to reduce inflammatory cytokines and transcription factor gene expression in macrophage cell culture [22,23,24]. In rats, *S. ebulus* fruits exert anti-inflammatory activity in a carrageenan-induced paw model [28]. The observed reduction in inflammatory cytokines in previous studies could be attributed to anthocyanins, ursolic acid, and other biologically active compounds found in *S. ebulus* fruits, which are rich in biologically active substances such as polyphenols, flavonoids, anthocyanins, phytosterols, flavonoids, triterpenes, tannins, iridoid glycosides, cardiac glycosides, derivatives of caffeic acid (such as volatile substances), chlorogenic acid, ursolic acid, and lectins [12,13,14,15,16,17]. Anthocyanins reach up to 25% of total polyphenols in *S. ebulus* fruit aqueous infusion [15]. In support of this speculation are findings about their anti-inflammatory activity [61,62,63]. *S. nigra* anthocyanin-rich extracts and preparations have been found to exert anti-inflammatory potential, decreasing expression levels of IL-1, IL-6, TNFα, and IL-8 in cultured primary [64] and RAW264.7 macrophages [65]. Recently, the anti-inflammatory potential of cyanidin-diglucosides was demonstrated in a cell culture model of inflammation. Significantly reduced levels of TNFα IL-1β, IL-6, and IL-8 were reported after cyanidin-3-O-glucoside treatment of LPS-stimulated macrophages [66].

The complement system (CS) is composed of a number of small proteins circulating in blood in its non-active precursor form. It is part of the innate non-adaptable immunity and becomes activated upon specific stimuli. The complement system is responsible for the stimulation of phagocytes to clear foreign and damaged cells, inflammation to attract additional phagocytes, and activation of the cell-killing membrane attack complex [67]. Thus, the complement system actively regulates various steps of an inflammatory response [68,69].

Keeping in mind that *S. ebulus* is used as an immunomodulatory remedy, we decided to determine the possible effect of *S. ebulus* fruit tea intake on complement system activity by measuring C3 and C4 levels in serum. Both C3 and C4 were significantly decreased in the whole group (Figure 1). Gender stratification showed a significant C4 decrease in women only. Decreased levels of the respective proteins may have two possible explanations. The first one is increased activation of the complement system leading to consumption of these proteins, which are components of the complement cascade. The other one implies a lowered C3 and C4 synthesis, and this hypothesis may be connected with the anti-inflammatory potential of the *S. ebulus* fruit tea. This suggestion seems reasonable, keeping in mind the high level of correlation between changes in the inflammatory parameters hs-CRP, TNFα, and C3 and C4 (Table 5). C3 synthesis is upregulated by various cytokines, including IL-1β and TNFα. Suppression of complement activity could be another possible mechanism behind the anti-inflammatory effects of *S. ebulus*. A similar link between anti-inflammatory and complement inhibitory activity was observed for glucocorticoids reported to suppress gene expression of C3, additionally to their inhibitory effect on the conversion of phospholipase A2 to arachidonic acid [70].

In fact, the complement system actively regulates various steps of the inflammatory response [68,69]. Complement activation involves Toll-like receptor (TLR) pathways triggered by inflammatory factors, such as CRP [68]. On the other hand, expression upregulation of inflammatory cytokines (IL-1β, IL-6, IL-8, and TNFα) involves the same receptor family [71,72,73]. NF-kB might have a crucial role in the observed anti-inflammatory and anti-complement activity of *S. ebulus* fruit tea, as it is responsible for TLR-mediated upregulation of pro-inflammatory molecules. Involvement of NF-kB regulation in observed results of *S. ebulus* intake in inflammation and the complement system is indirectly supported by the observation in rats where, although nonsignificantly, *S. nigra* supplementation decreased NF-kB in rat liver [74]. In cell cultures (J774A.1 macrophages), *S. ebulus* fruit extracts prevented LPS-induced NF-kB and related gene expression [22]. In the same model, SE total extract and anthocyanin enriched fraction appeared to prevent LPS induction of TLR4 transcription and related downstream proteins and other inflammation-related molecules (TNFα, IL-1β, IL-6, COX-2, inducible NOS, IL-1RN, MCP-1, and CRP) [24].

In addition to the well-known cytokine-inhibiting properties, bioflavonoids extracted from medicinal plants are reported to inhibit also the complement activity in vitro [63,75].

A third assumption may relate decreased complement components to improved border immunity, indirectly leading to lower inflammatory status. For example, polyphenols have been reported to regulate the intestinal mucosal immune response [76].

Complement fixation activity has been established for *S. nigra* pectins [77]. Using this model, Cos et al. [78] find the inhibitory activity of forty-two ethanolic extracts of thirty-six Rwandan medicinal plants, and it has been established that this is not related to the chelation activity or direct action on the target erythrocytes. In vitro complement activity fixation properties have also been established for plant polysaccharides [79] and glycoconjugates [80]. Jia and coauthors [81] determined the complement in vitro inhibitory activity of extracts and isolated compounds from *Eucommia ulmoides*. In contrast, Nhu et al. [82] established the stimulatory effect of 20 medicinal plants on complement activation in cultured fish PBMC and head kidney leucocytes. Studying *Tamarindus indica* extract, Landi Librandi and coauthors [83] establish in vitro stimulation of the classical/lectin pathway and the inhibition of alternative pathways. The same extract, when administered in vivo, blocked the increase in complement activity caused by a cholesterol-rich diet decreasing the lytic activity of serum in hamsters. In fish, dietary tea polyphenols decreased both C3 and C4, and this was also associated with the decrease in inflammatory factors IL-1, IL-6, and TNFα [84]. The same study suggested a possible involvement of gut microbiota in these effects.

In the present study, blood cell count was undertaken in an effort to determine the possible effect of *S. ebulus* fruit tea intake on host defense and hematopoiesis. No significant change in all of the studied parameters was established, though with a borderline significance, an increase in the percentage of lymphocytes was established in the whole group by 1.91% and in the women group by 5.73%, *p* < 0.05 (Table 2 and Table 3). We suppose that the changes are primarily relative and correspond to decreased total counts and percentage of neutrophils, monocytes, and eosinophils, respectively, which also remain nonsignificantly changed. Similar results were obtained by Salvador and coauthors [85], where dietary supplementation of rats with elderberry polar and lipophilic extracts resulted in a significant increase in lymphocytes. They established a significant increase in whole WBC fraction and also in monocytes, as well, which were nonsignificantly decreased in our study.

A very small, but still significant, decrease in hemoglobin (1.20%, *p* < 0.05) and hematocrit (1.55%, *p* < 0.01) in the whole group after the intervention was detected (Table 2). Gender subgrouping showed that these parameters were decreased in the women group only (1.61%, *p* < 0.01 and 2.20, *p* < 0.001, respectively). A slight nonsignificant decrease in RBC was also detected in the women group. In order to check whether the menstrual cycle would be involved in this result, we created and analyzed another group composed of males and women in menopause. We measured their RBC, Hb, and hematocrit levels before and after the *S. ebulus* intervention (Figure 1). No changes in these parameters were detected in this group, representing that these results may be due to menstrual events during the intervention period.

A significant positive correlation was observed between changes (∆) in hs-CRP and total leucocytes (WBC), accompanied by a significant negative correlation between ∆hs-CRP and the ∆lymphocytes (%). This may be explained by the fact that higher levels of the pro-inflammatory hs-CRP mobilizes more leucocytes, especially neutrophils and monocytes, to the inflammation area, decreasing the percentages of lymphocytes triggered by the innate immune response. We can make a hypothesis that *S. ebulus* fruit intake may contribute to some kind of immunomodulation, increasing the percentage of lymphocytes and probably leading to the activation of adaptive immunity. Prior to the intervention with *S. ebulus*, no significant correlations were observed between TNFα and the total of leucocytes (WBC), nor between TNFα and the percentages of lymphocytes.

## 4. Materials and Methods

### 4.1. Plant Material and Infusion Preparation

The plant material used in the study was a commercial product supplied from the local markets, packaged by Medicam Ltd., Varna, Bulgaria, with guaranteed origin and botanical identification prior to plant processing.

Dried fruits from *Sambucus ebulus* were used for infusion preparation. The recipe was adapted from Bulgarian folk medicine; specifically, 2.5 g of plant material was added to 300 mL of boiled water and was incubated for 30 min. The infusion was filtered and used for phytochemical analyses or for human intervention.

### 4.2. Phytochemical Analysis

The preparation of the samples using Solid Phase Extraction (SPE) method and the subsequent analyses through UPLC-ESI-MS/MS (LTQ Orbitrap mass spectrometer, Thermo Scientific, Hemel Hempstead, UK) in negative mode were performed as described in [18]. Quantification of anthocyanins was performed by the external standard method using the standards indicated in Table 6, also representing performance parameters of the LC-MS methodology.

### 4.3. Subjects

A group of 53 healthy volunteers (men/women = 9/43, age 20–60) underwent the intervention with *S. ebulus* fruit infusion for a period of 4 weeks. Exclusion criteria were chronic diseases (diabetes, renal, liver, gastrointestinal, cardiovascular, autoimmune, oncological diseases, hypertonia, and psychiatric disturbances), acute inflammatory conditions at the moment of the intervention, pregnancy, lactation, and taking specified therapeutics, including homeopathic drugs and immunomodulatory supplements. In the course of intervention, two subjects dropped out due to illness, and 51 subjects (9 men and 41 women) completed the study.

### 4.4. Ethical Issues

The study was approved by the Research Ethics Committee at the Medical University of Varna (Protocol No 85/26.07.2019). Before entering the study, all subjects signed an informed consent.

### 4.5. Study Design

The intervention study included intake of 200 mL infusion, prepared every morning (between 8.30 and 9.00 a.m.) immediately before the consumption for a period of 4 weeks. Furthermore, participants were strongly recommended not to change their dietary habits and lifestyle throughout the intervention period. Fasting blood samples were collected in heparin vacutainers at the beginning (T1) and at the end (T2) of the intervention study. Serum aliquots were stored at −80 °C until analyses.

### 4.6. Blood Cells Count

Complete blood count of 22 indicators + ESR (WBC, NEO%, LYM%, MONO%, EOS%, BASO%, NEO #, LYM #, MONO #, EOS #, BASO #, RBC, HGB, HCT, MCV, MCH, MCHC, RDW, PLT, MPV, PCT, and PDW) was performed on Sysmex XN-1000™ Hematology Analyzer (Kobe, Japan), using a laser-optic counting by flow cytometry for leucocytes, and hydrodynamic focusing with DC detection for erythrocytes and thrombocytes.

### 4.7. Protein Levels Measurement

For analysis of IL-1β, IL-6, and TNFα in serum samples, we used Siemens IMMULITE 1000 system and their respective kits (IMMUL/1000 IL-1, IMMUL/1000 IL-6, IMMUL/1000 TNF-a). All 3 tests are with ISO 9001:2008 and CE mark. For analysis of CRP, we used Beckman-Coulter AU640 system with their latex CRP kit (OSR6199). Enzyme-linked immunosorbent assay (ELISA) was applied for determination of plasma concentration of IL-8 (Human IL-8 ELISA kit, Diaclone) using manufacturer’s protocol. Absorbance was measured on Synergy 2 plate reader (BioTek). Components of complement C3 and C4 were also analyzed with Beckman-Coulter C3 (OSR6159) and C4 (OSR6160) kits on Beckman-Coulter AU640 instrument. Total serum protein levels were measured with Beckman-Coulter TP kit on Beckman-Coulter AU640 instrument.

### 4.8. Statistical Analyses

The statistical analysis was carried out using GraphPad Prism 6.0 software. Quantitative data were expressed as mean ± SEM. Paired-samples t-test was applied to compare the T1 and T2 markers for the total group. Spearman correlation analysis was used to evaluate the causal links between the tested parameters. Statistical significance was considered at *p* < 0.05.

## 5. Conclusions

In conclusion, *Sambucus ebulus* fruit infusion exerts immune-modulatory activity, as revealed by decreased pro-inflammatory status and complement activity markers in healthy volunteers. On the basis of the data obtained, a lowered defense power in case of subsequent infectious disease could be expected. Further investigations would elucidate elderberries’ potential as a preventive and healing remedy in case of infection. We believe that these results will be valuable for the aims of functional foods and nutraceuticals development, given the higher content of bioactives presented by anthocyanins, where cyanidin-3-O-galactoside and cyanidin 3-sambubioside were the most abundant.

## Figures and Tables

**Table 1:** Anthocyanin content in *S. ebulus* fruit infusion.

#	Compound	UVmax	Molecular Formula [M-H]^−^	[M-H]^−^ m/z	MS/MS Ions	Mean ± SD [µg/mL]	Mean ± SD [mg/g DW]
1	Cyanidin-3-O-galactoside	519, 279	C21H21O11	449.12	287.05, 271.05, 227.03	401.26 ± 15.01	48.16 ± 1.8
2	Cyanidin-3-O-glucoside	516, 280	C21H21O11	449.12	287.06, 271.06, 227.03	32.62 ± 1.22	3.91 ± 0.14
3	Cyanidin-3-O-arabinoside	530	C20H19O10	463.11	287.07, 257.11, 229.02	90.16 ± 3.37	10.82 ± 0.40
4	Cyanidin-3-O-xyloside	520	C20H19O10	447.10	287.18, 271.00, 245.22	15.07 ± 0.56	1.81 ± 0.07
5	Cyanidin 3,5-diglucoside	518, 284	C27H31O16	611.16	449.06, 287.05, 271.05, 151.02	38.65 ± 6.12	4.64 ± 0.73
6	Cyanidin 3-sambubioside	518, 280	C27H33O16	581.11	419.08, 287.06, 271.06, 151.02	361.79 ± 8.91	43.41 ± 1.07

**Table 2:** Gender stratification of biochemical and hematological parameters before and after intervention with *S. ebulus*. T1 (before intervention); T2 (after intervention); ∆ (change). *p* value was calculated using paired *t*-test.

Women	n	T1 (Mean ± SD)	T2 (Mean ± SD)	Δ (T2-T1) (Mean ± SD)	Δ (T2-T1) (%)	*p*
Total protein (g/L)	42	73.51 ± 4.26	71.22 ± 4.65	−2.28 ± 3.89	−3.11	<0.001
IL-8 (pg/mL)	33	14.89 ± 2.31	14.18 ± 2.38	−0.71 ± 1.99	−4.76	<0.05
TNFα (pg/mL)	36	6.28 ± 1.09	5.96 ± 1.03	−0.31 ± 0.83	−5.09	<0.05
C3 (g/L)	42	0.93 ± 0.16	0.90 ± 0.16	−0.03 ± 0.11	−3.22	=0.06
C4 (g/L)	42	0.27 ± 0.08	0.24 ± 0.072	−0.03 ± 0.04	−11.11	<0.0001
Lymphocytes (%)	42	33.31 ± 7.62	35.22 ± 7.72	1.91 ± 6.27	+5.73	<0.05
RBC (×10^12^/L)	42	4.52 ± 0.34	4.48 ± 0.34	−0.04 ± 0.15	−0.88	=0.07
Hb (g/L)	42	130.20 ± 8.72	128.10 ± 8.95	−2.04 ± 4.51	−1.61	<0.01
Hematocrit (L/L)	42	0.409 ± 0.023	0.400 ± 0.023	−0.0083 ± 0.014	−2.20	<0.001
**Men**	**n**	**T1** **(Mean ± SD)**	**T2** **(Mean ± SD)**	**Δ (T2-T1)** **(Mean ± SD)**	**Δ (%)**	***p***
IL-6 (pg/mL)	6	4.26 ± 1.24	2.53 ± 0.48	−1.73 ± 1.24	−40.61	<0.05
C4 (g/L)	9	0.29 ± 0.07	0.22 ± 0.09	−0.06 ± 0.09	−24.13	=0.07

IL-6—interleukin 6; IL-8—interleukin 8; TNFα—tumor necrosis factor alpha; RBC—red blood cells; Hb—hemoglobin.

**Table 3:** Hematological parameters before and after intervention with *S. ebulus* fruit tea. T1 (before intervention); T2 (after intervention); and ∆ (change). *p* value was calculated using paired *t*-test.

Parameters	n	T1 (Mean ± SD)	T2 (Mean ± SD)	Δ (T2-T1) (Mean ± SD)	Δ (T2-T1) (%)	*p*
WBC (×10^9^/L)	51	6.42 ± 1.54	6.28 ± 1.68	−0.14 ± 1.27	−2.18	ns
Neutrophils (%)	51	53.91 ± 8.77	52.22 ± 9.19	−1.68 ± 7.69	−3.13	ns
Neutrophils (×10^9^/L)	51	3.54 ± 1.31	3.38 ± 1.49	−0.15 ± 1.20	−4.51	ns
Lymphocytes (%)	51	33.62 ± 7.60	35.39 ± 8.04	1.77 ± 6.91	5.26	=0.07
Lymphocytes (×10^9^/L)	51	2.09 ± 0.49	2.13 ± 0.48	0.04 ± 0.40	1.91	ns
Monocytes (%)	51	8.63 ± 2.06	8.55 ± 1.74	−0.07 ± 1.54	−0.92	ns
Monocytes (×10^9^/L)	51	0.54 ± 0.15	0.52 ± 0.12	−0.02 ± 0.12	−3.70	ns
Eosinophils (%)	51	2.97 ± 2.10	2.91 ± 1.83	−0.06 ± 1.44	−2.02	ns
Eosinophils (×10^9^/L)	51	0.19 ± 0.16	0.17 ± 0.10	−0.01 ± 0.11	−10.52	ns
Basophils (%)	51	0.86 ± 0.41	0.91 ± 0.41	0.05 ± 0.23	5.81	ns
Basophils (×10^9^/L)	51	0.056 ± 0.03	0.06 ± 0.02	−0.001 ± 0.02	−1.78	ns
RBC (×10^12^/L)	51	4.64 ± 0.43	4.61 ± 0.46	−0.03 ± 0.17	−0.64	ns
Hb (g/L)	51	133.30 ± 11.20	131.70 ± 12.14	−1.21 ± 5.72	−1.20	<0.05
Hematocrit (L/L)	51	0.41 ± 0.03	0.42 ± 0.03	−0.01 ± 0.02	−1.55	<0.01
Thrombocytes (×10^9^/L)	51	271.40 ± 63.13	270.50 ± 58.70	−0.86 ± 29.47	−0.33	ns

WBC*—*white blood cells; RBC*—*red blood cells; Hb*—*hemoglobin; and ns*—*nonsignificant (*p* > 0.05).

**Table 4:** Hematological parameters before and after intervention with *S. ebulus* fruit tea for men/women in menopause and fertile women. T1 (before intervention); T2 (after intervention); ∆ (change). *p* value was calculated using paired *t*-test.

Men and Women in Menopause	n	T1 (Mean ± SD)	T2 (Mean ± SD)	Δ (T2-T1) (Mean ± SD)	Δ (T2-T1) (%)	*p*
RBC (×10^12^/L)	20	4.86 ± 0.42	4.86 ± 0.48	0.00 ± 0.06	0.00	ns
Hb (g/L)	20	142.00 ± 11.97	142.30 ± 15.02	−0.30 ± 3.05	0.21	ns
Hematocrit (L/L)	20	0.43 ± 0.02	0.43 ± 0.03	0.00 ± 0.01	0.00	ns
**Women in Fertile Age**	**n**	**T1 ** **(Mean ± SD)**	**T2 ** **(Mean ± SD)**	**Δ (T2-T1)** **(Mean ± SD)**	**Δ (T2-T1) (%)**	***p***
RBC (×10^12^/L)	31	4.52 ± 0.36	4.47 ± 0.38	−0.05 ± 0.02	−1.10	ns
Hb (g/L)	31	129.70 ± 8.09	127.40 ± 9.06	−2.30 ± 0.97	−1.77	<0.05
Hematocrit (L/L)	31	0.41 ± 0.02	0.40 ± 0.03	−0.01 ± 0.00	−2.00	<0.01

RBC*—*red blood cells; Hb*—*hemoglobin; ns*—*nonsignificant (*p* > 0.05).

**Table 5:** Correlation analysis between changes in inflammatory cytokines and C3 and C4 (Δ (T2-T1) and all the other studied parameters.

∆IL-6/	r	*p*
∆IL-8	−0.56	<0.05
∆WBC	0.48	<0.05
∆neutrophils ×10^9^/L	0.46	<0.05
∆RBC	−0.32	<0.05
∆Hb	−0.30	<0.05
hematocrit	−0.38	<0.05
**∆TNFα/**	**r**	***p***
∆C4	0.31	<0.05
∆eosinophils (%)	0.32	<0.05
∆eosinophils ×10^9^/L	=0.33	<0.05
**∆hs-CRP/**	**r**	***p***
∆TNFα	0.35	<0.05
∆C3	0.44	<0.01
∆C4	0.52	<0.0001
∆WBC	0.46	<0.001
∆neutrophils ×10^9^/L	0.47	<0.001
∆lymphocytes (%)	−0.42	<0.01
∆monocytes	0.46	<0.001
∆eosinophils ×10^9^/L	0.32	<0.05
**∆C3/**	**r**	***p***
∆C4	0.8	<0.0001
∆total protein	0.49	<0.01
∆RBC	0.50	<0.001
Hb	0.31	<0.05
hematocrit	0.38	<0.01
**∆C4/**	**r**	***p***
∆lymphocytes ×10^9^/L	0.27	=0.05
RBC	0.38	<0.01
∆thrombocytes	0.27	<0.05

hs-CRP*—*high sensitivity C-reactive protein; IL-6*—*interleukin 6; IL-8*—*interleukin 8; TNFα*—*tumor necrosis factor alpha; WBC*—*white blood cells; RBC*—*red blood cells; and Hb*—*hemoglobin.

**Table 6:** Performance parameters of the LC-MC methodology.

Standard	Linear Range (ng/mL)	LOQ (ng/mL)	LOD (ng/mL)	Recovery (%)	Precision (RSD, %)		Repeatability
					Intra-Day	Inter-Day	(RSD, %, n = 6)
cyanidin-3-O-glucoside	1.18–960	1.18	0.40	94.50 ± 1.45	2.20	3.11	2.77
cyanidin 3,5-diglucoside	1.36–1155	1.36	0.66	88.65 ± 1.75	1.77	2.66	3.66
cyanidin 3-sambubioside	0.98–1211	0.98	0.30	82.44 ± 1.88	3.11	4.00	4.40

LOD: limit of detection*;* LOQ: limit of quantification.

## Data Availability

The authors confirm that the data supporting the findings of this study are available within the article.
